# The emerging role of autophagy in peroxisome dynamics and lipid metabolism of phyllosphere microorganisms

**DOI:** 10.3389/fpls.2014.00081

**Published:** 2014-03-11

**Authors:** Masahide Oku, Yoshitaka Takano, Yasuyoshi Sakai

**Affiliations:** ^1^Division of Applied Life Sciences, Graduate School of Agriculture, Kyoto UniversityKyoto, Japan; ^2^Division of Applied Biosciences, Graduate School of Agriculture, Kyoto UniversityKyoto, Japan; ^3^Research Unit for Physiological Chemistry, Center for the Promotion of Interdisciplinary Education and Research, Kyoto UniversityKyoto, Japan

**Keywords:** autophagy, lipid droplet, methylotrophic yeast, peroxisome, phyllosphere, phytopathogenic fungus

## Abstract

Eukaryotic microorganisms resident in the phyllosphere (above-ground, plant-surface environments) undergo dynamic changes in nutrient conditions and adapt their metabolic pathways during proliferation or in the course of infection of host plants. Some of these metabolic switches are accomplished by regulation of organelle abundance. Recent studies have shown that autophagy plays a major role in reducing the organelle quantity, thereby contributing to the metabolic switch required for survival or virulence of the microorganisms in the phyllosphere. In this mini review the metabolic pathways in several phytopathogenic fungi and the non-infectious asporogenous yeast *Candida boidinii*, which involve lipid droplets and peroxisomes, are summarized. The physiological functions of Atg (Autophagy-related) proteins in these organisms are discussed in relation to the dynamics of these two important organelles.

## INTRODUCTION

**FIGURE 1:**
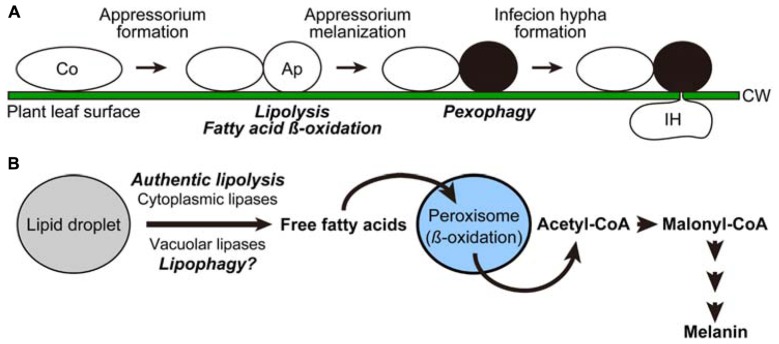
**Cellular and metabolic remodeling processes in the phytopathogenetic fungus *Colletotrichum orbiculare*. (A)** Cell differentiation processes of *Colletotrichum orbiculare*. Intracellular dynamics induced at the transition steps are indicated in italics. Ap, appressorium; Co, conidium; CW, cell wall; IH, infection hypha. **(B)** Model scheme of the metabolic flow induced for melanin biosynthesis.

The phyllosphere has been under evaluated as an ecological site for plant-microbe interaction, compared with the rhizosphere (Vorholt, 2012). However, dynamic environmental changes, i.e., light, heat, nutrient, plant immunity response, should affect the life style of microbes in the phyllosphere. Among the eukaryotic inhabitants, only plant-infectious fungi have been the subject of extensive studies based on their important influences on crop yields (Agrios, 2004; Dean et al., 2012). In general, asexual spores (conidia) of phytopathogenic fungi undergo sequential cellular differentiation on the plant-surface, i.e., conidia germinate and germ tubes of conidia differentiate into a specific cellular apparatus termed the appressorium for host invasion (**Figure 1A**). The process of differentiation requires induction/inactivation of specific metabolic pathways. In the following section of this mini review, we first introduce the melanin biosynthesis pathway, which is conserved among a subset of phytopathogenic fungi and is closely associated with the dynamics of lipid droplets and peroxisomes. Then several functional links between this biosynthetic pathway and autophagy are discussed.

Recent studies revealed that non-phytopathogenic eukaryotic microorganisms can also establish an inhabitation in the phyllosphere. One such example is the asporogenous methylotrophic yeast *Candida boidinii*, which can grow on methanol as a sole carbon and energy source (Kawaguchi et al., 2011). Although *Candida boidinii* could not form stress-resistant spores, this organism is often isolated from plant-surfaces, and was shown to proliferate on the leaves of growing *Arabidopsis thaliana*. Furthermore, phyllospheric growth depended on methanol, whose concentration fluctuated during the daily light–dark cycle. As a result of methanol fluctuation, *Candida boidinii* increased or decreased its quantity of peroxisomes. In the last section of this mini review, the function of peroxisome-specific autophagy (termed pexophagy), which is vital for the decrease of peroxisome quantity and for the growth of this organism in the phyllosphere, is summarized.

## PEROXISOME AND LIPID DROPLET DYNAMICS IN PHYTOPATHOGENIC FUNGI

### PEROXISOME AND LIPID DROPLET DYNAMICS FOR MELANIN BIOSYNTHESIS PATHWAY

Several genera of phytopathogenic fungi, including *Colletotrichum* and *Magnaporthe* species, develop appressoria pigmented with melanin for efficient infection of their host plants (Kubo and Furusawa, 1991). The melanin layer in the appressorial cell wall is thought to determine the correct penetration site because melanin is excluded from this site (Kubo and Furusawa, 1991). Melanin also provides the rigidity and selective permeability of the appressorial cell wall, and is considered to be responsible for generating the turgor pressure required for the appressorial penetration (Howard and Ferrari, 1989; Bechinger et al., 1999).

Since the precursor compound for melanin synthesis, malonyl-CoA, was found to be derived from acetyl-CoA in *Colletotrichum orbiculare* (Syn. *Colletotrichum lagenarium*; Asakura et al., 2012), robust biosynthesis of melanin in this organisms depends on mobilization of free fatty acids from lipid droplets, followed by the conversion of the liberated lipids into acetyl-CoA through the beta-oxidation pathway in peroxisomes (**Figure 1B**). Consistent with this, it was shown that functional assembly of peroxisomes was required for the formation of melanized appressoria as well as the pathogenicity of *Colletotrichum orbiculare *(Kimura et al., 2001).

Our recent study demonstrated that the liberation of fatty acids from lipid droplets (termed lipolysis) in the appressorium was inhibited by blocking either the (1) beta-oxidation pathway, (2) conversion of acetyl-CoA to malonyl-CoA, or (3) consumption of malonyl-CoA by melanin biosynthesis (Asakura et al., 2012). This indicates that an intact melanin biosynthesis pathway is required for lipolysis (**Figure 1B**). Molecular details underlying this feedback regulation are not clear, but it is possible that the intermediates of the pathway, namely free fatty acids, acetyl-CoA, and/or malonyl-CoA, might act as signaling molecules to repress lipolysis, in order to avoid the deleterious effect called “lipotoxicity,” which results from excess accumulation of lipid substances, especially free fatty acids. In many experimental systems including yeast, the formation of lipid droplets is strongly suggested to contribute to the prevention of lipotoxicity by incorporating free fatty acids into the core contents of the organelle, or into neutral lipids (Eisenberg and Buttner, 2013).

The precise mechanism underlying the induction of lipolysis during the appressorium melanization has not been fully elucidated. An authentic definition of lipolysis solely refers to cleavage of neutral lipids inside lipid droplets by the action of cytoplasmic lipases (Zechner et al., 2012). In a mammalian experimental system, this reaction is known to be activated by the protein kinase A (PKA)-mediated signaling pathway (Holm, 2003), which is also known to be important for up-regulating the lipolysis activity and pathogenicity in several phytopathogenic fungi (*Magnaporthe oryzae* and *Colletotrichum orbiculare*; Thines et al., 2000; Yamauchi et al., 2004). Furthermore, a pioneering study on the morphological details of appressorium formation in *M. oryzae *demonstrated that lipid droplets are incorporated into the vacuolar portion of the cell and are degraded therein (Weber et al., 2001), which fits the fundamental criterion of lipid droplet autophagy (lipophagy). In this case, lipases inside the vacuole, not those in the cytoplasm, are thought to be responsible for the breakdown of neutral lipids, but they remain to be identified. Although the microautophagic process (a type of autophagy that includes direct engulfment of the target organelle by vacuolar/lysosomal membrane) was suggested to be involved in lipid droplet degradation in *Saccharomyces cerevisiae* (van Zutphen et al., 2014), little is known about the molecular details of lipophagy, especially in terms of the mechanism targeting lipid droplets to the vacuole. Uncovering the factors functioning in lipophagy will be of great value for understanding how these different modes of lipolytic activities (authentic lipolysis and lipophagy) are utilized in phytopathogenic fungi.

### PEXOPHAGY REQUIRED FOR INFECTION OF *Colletotrichum orbiculare*

After formation of melanized appressoria in *Colletotrichum orbiculare*, the peroxisomes therein are subjected to degradation. Morphological experiments showed that the peroxisomes to be degraded were encapsulated by a membrane structure labeled with Atg8, and transferred into the lumen of the vacuole, showing a typical pattern of peroxisome-specific autophagy (pexophagy; Asakura et al., 2009). Consistent with this observation, the Atg26 protein, which was previously shown to be specifically required for pexophagy in the methylotrophic yeast *Pichia pastoris *(See next section; Oku et al., 2003), was found to be necessary for degradation of peroxisomes in the appressorium. Loss of Atg26 in *Colletotrichum orbiculare* abolished the functionality of the appressorium for the host plant invasion and thus impaired pathogenicity to the host. Notably, the appressorium formed by the *atg26 *mutant strain was more resistant to hyper-osmotic shock than that formed by the wild-type strain, implying that pexophagy affects cell wall integrity or stiffness of the phytopathogenic apparatus for the process of penetration during the course of infection.

## PEROXISOME DYNAMICS IN THE METHYLOTROPHIC YEAST RESIDENT ON PLANT LEAVES

### MOLECULAR MACHINERY OF PEXOPHAGY IN METHYLOTROPHIC YEASTS

A striking feature of methylotrophic yeasts is that they develop numerous peroxisomes that contain several primary enzymes in the methanol-metabolizing pathway, such as alcohol oxidase (Aod) and dihydroxyacetone synthase (Das), when these organisms are grown on methanol (Yurimoto et al., 2011). Owing to this property, the methylotrophic yeasts have been considered to be good model organisms to study peroxisome dynamics, including pexophagy (Oku and Sakai, 2010).

Insights gained from these organisms include the molecular requirements for pexophagy of the so-called “core” Atg proteins for *de novo* membrane biogenesis (Yang and Klionsky, 2010), and the identification of several pexophagy-specific factors associated with these core proteins. One of the pexophagy-specific factors identified in *P. pastoris* is Atg26. This protein, encoding a sterol glucosyltransferase, was found to be required for degradation of methanol-induced peroxisomes (Nazarko et al., 2007), but to be dispensable for macroautophagy induced by nitrogen-source starvation (Oku et al., 2003). This protein was shown to act downstream of the phosphatidylinositol 4’-kinase (PI4K) signaling pathway, and was localized to the pre-autophagosomal structure (PAS; Yamashita et al., 2006). Another pexophagy-specific factor, Atg30, was identified as a receptor molecule on peroxisomes recognized by several core Atg proteins (Farre et al., 2008). The interactions between Atg30 and core Atg proteins (Atg8 and Atg11) were dependent on phosphorylation of two serine residues within Atg30 (Farre et al., 2013). By utilizing strains mutated in or devoid of these pexophagy-specific proteins, we are now able to reveal the physiological functions of pexophagy separately from the roles of general (bulk) autophagy.

### METHANOL METABOLISM AND AUTOPHAGY IN *Candida boidinii* REQUIRED FOR GROWTH ON PLANT LEAVES

**FIGURE 2:**
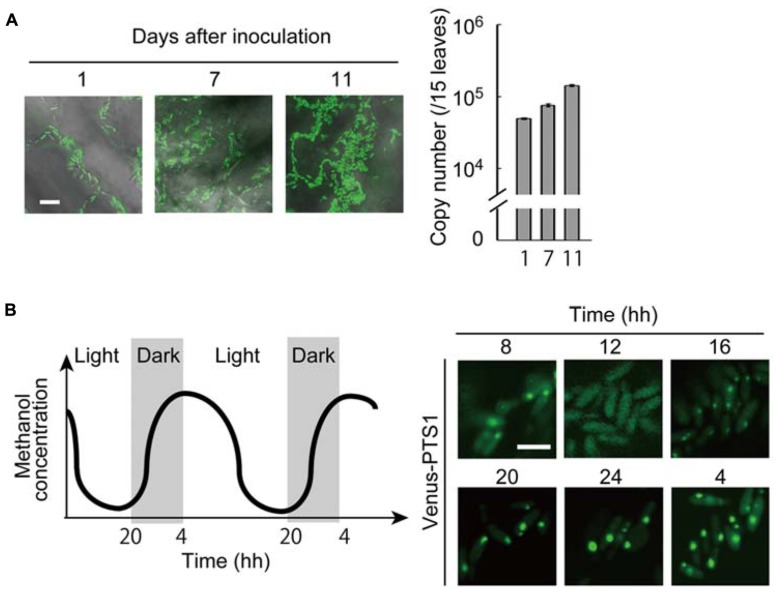
**Peroxisome dynamics in the methylotrophic yeast *Candida boidinii *in the phyllosphere.** All of the experimental data were reconstructed from those in the study by Kawaguchi et al. (2011). **(A)** (Left) Microscopic images of the *Arabidopsis thaliana* leaf surface with fluorescent *Candida boidinii* cells. After inoculation with the yeast cells, the plant was grown for the designated number of days in a chamber equipped with an illumination system to generate a daily light–dark cycle. Bar, 10 μm. (Right) Results of quantitative PCR using the plant leaf samples obtained after the designated number of days after yeast inoculation. The copy numbers indicate those of the *VENUS* gene integrated in the genome of the inoculated *Candida boidinii* cells, and thus represent growth of the inoculated *Candida boidinii* cells. The error bars show standard deviations. **(B)** (Left) Schematic drawing of periodical changes in methanol concentrations on *A. thaliana* leaves. (Right) At the indicated times, peroxisomes in the *Candida boidinii* cells resident on *A. thaliana *leaves were visualized with Venus harboring a peroxisome targeting signal (PTS) 1. Bar, 5 μm.

Our recent study took advantage of the expression of a fluorescent protein (Venus) for the enhanced, quantitative detection of *Candida boidinii* cells inoculated on *A. thaliana* leaves (Kawaguchi et al., 2011). Through this technique we revealed that the inoculated *Candida boidinii *cells were able to proliferate on the leaf surface of plants grown in a chamber with illumination generating a daily light–dark cycle (**Figure 2A**). Growth was dependent on its methanol-metabolizing enzymes, Aod1 and Das1. In addition, methanol concentrations available to the organism on the plant leaves were also determined with the fluorescence intensity of Venus expressed under the regulation of a methanol-inducible promoter. Interestingly, dynamic oscillation of methanol concentration was observed on the surface of *A. thaliana* leaves during the daily light–dark cycle, with higher concentrations in the dark period (**Figure 2B**). In accordance with this oscillation, peroxisome abundance in the inoculated *Candida boidinii *cells was found to fluctuate as revealed by fluorescence microscopy of a peroxisome-targeted Venus: the organelle quantity increased in the dark period, and decreased in the light period (**Figure 2B**). We assume that the methanol-induced peroxisome serves as a storage organelle for proteins to replenish amino-acid pools needed in the natural environment, in order for the immotile microorganism to survive until they obtain nutrients for further proliferation.

Since *Candida boidinii *possessed a homolog of *P. pastoris* Atg30 (the pexophagy-specific protein), we inoculated the *Cbatg30Δ* strain on plant leaves. The decrease in peroxisome quantity in the light period was suppressed by the loss of Atg30. Notably, this mutant strain exhibited a severe growth defect on plant leaves, similar to mutant strains incapable of methanol metabolism. The transport of Atg8 into the vacuole, a marker of general autophagy was detected in the inoculated* Candida boidinii Cbatg30*Δ strain, but not in the *Cbatg1*Δ strain, indicating that general autophagy occurs throughout the day. However, it is still unknown how autophagic activity and selectivity are regulated along the daily light–dark cycle.

## CONCLUDING REMARKS

Despite great advances in the understanding of the molecular mechanism of pexophagy, the physiological role of this selective autophagy had been an enigma for a long time, until the microorganisms were transferred from laboratory media to plant-surfaces or plant surface-mimicking environments. Likewise, the dynamics of lipid droplets is now an exciting topic of cell biology, and studies of eukaryotic microorganisms in the phyllosphere will give us profound insight into the physiological importance of organelle dynamics. Accumulating molecular information on the microorganisms in the phyllosphere will also be important for a better understanding of plant-microorganism interactions.

## Conflict of Interest Statement

The authors declare that the research was conducted in the absence of any commercial or financial relationships that could be construed as a potential conflict of interest.
